# Patient-derived hepatitis C virus inhibits CD4^+^ but not CD8^+^ T lymphocyte proliferation in primary T cells

**DOI:** 10.1186/s12985-015-0322-4

**Published:** 2015-06-19

**Authors:** Sonya A. MacParland, Annie Y. Chen, Christopher P. Corkum, Tram N.Q. Pham, Tomasz I. Michalak

**Affiliations:** Molecular Virology and Hepatology Research Group, Division of BioMedical Sciences, Faculty of Medicine, Health Sciences Centre, Memorial University, St. John’s, Newfoundland and Labrador Canada; Present address: Department of Immunology, Medical Sciences Building, University of Toronto, Toronto, ON Canada; Present address: Laboratory of Human Retrovirology, Institut de Recherches Cliniques de Montreal (IRCM), Montreal, QC Canada

**Keywords:** HCV, HCV lymphotropism, HCV infection of T cells, T cell proliferation, T cell apoptosis, T cell cytokine expression

## Abstract

**Background:**

Hepatitis C virus (HCV) can replicate in cells of the immune system and productively propagate in primary T lymphocytes in vitro. We aimed to determine whether exposure to authentic, patient-derived HCV can modify the proliferation capacity, susceptibility to apoptosis and phenotype of T cells.

**Methods:**

Primary total T cells from a healthy donor were used as targets and plasma-derived HCV from patients with chronic hepatitis C served as inocula. T cell phenotype was determined prior to and at different time points after exposure to HCV. T cell proliferation and apoptosis were measured by flow cytometry-based assays.

**Results:**

The HCV inocula that induced the highest intracellular expression of HCV also caused a greatest shift in the T cell phenotype from predominantly CD4-positive to CD8-positive. This shift was associated with inhibition of CD4+ but not CD8+ T cell proliferation and did not coincide with altered apoptotic death of either cell subset.

**Conclusions:**

The data obtained imply that exposure to native HCV can have an impact on the relative frequencies of CD4+ and CD8+ T cells by selectively suppressing CD4^+^ T lymphocyte proliferation and this may occur in both the presence and the absence of measurable HCV replication in these cells. If the virus exerts a similar effect in vivo, it may contribute to the impairment of virus-specific T cell response by altering cooperation between immune cell subsets.

**Electronic supplementary material:**

The online version of this article (doi:10.1186/s12985-015-0322-4) contains supplementary material, which is available to authorized users.

## Introduction

Hepatitis C virus (HCV) is a single-stranded RNA virus that affects 130–150 million people worldwide as a chronic infection [1]. In HCV-infected patients, viral replication has been demonstrated not only in hepatocytes but also in cells of the immune system, such as B cells, monocytes, and CD4^+^ and CD8^+^ T lymphocytes [2–6]. In this laboratory, it was uncovered that primary T lymphocyte cultures, generated by ex vivo treatment of peripheral blood mononuclear cells (PBMC) from healthy individuals with a T cell-inducing mitogen phytoheamagglutin (PHA), are susceptible to wild-type (patient-derived) HCV and capable of supporting its replication at a level comparable to that of in vivo infected lymphoid cells [6, 7]. Furthermore, these cells were able to produce infectious virions that de novo infected lymphocytes [7]. It was also uncovered that patient-derived HCV is significantly more infectious to primary T cells than laboratory-derived clonal strains of HCV [8]. As well, HCV infection of T cells requires surface expression of CD5, and transfection of HCV non-susceptible T cell lines with CD5 renders these cells susceptible to infection [9].

HCV infection causes chronic hepatitis C (CHC) in up to 85 % of those afflicted, while acute HCV infection is thought to spontaneously resolve in 15-25 % of cases [1]. Resolution of hepatitis C appears to be a result of a robust HCV-specific T cell-mediated response. In HCV-infected chimpanzees, representing the closest animal model of human HCV infection, recovery from hepatitis C and a drop in plasma HCV loads to levels undetectable by clinical assays requires the activity of both CD4^+^ T helper cells and CD8^+^ cytotoxic T cells [10, 11]. In patients with CHC, T cells display markers of exhaustion and are defective in their ability to produce interferon-γ (IFN-γ) and interleukin-2 (IL-2) [12]. In vitro, the functional consequences of T cell infection in Molt-4 and Jurkat T cell lines and primary T cells have been investigated only using laboratory-adapted clonal strains of HCV [4, 13, 14]. It was found, among others, that infection with a HCV SB strain of Molt-4 cells suppressed IFN-γ signalling through the STAT-1 pathway [4]. This virus strain was also able to infect primary CD4^+^ T cells and this infection was associated with a decrease in their proliferation [14].

Stemming from the previous observation that infection of naïve lymphoid cells with patient-derived HCV can lead to changes in the CD4^+^ to CD8^+^ T cell ratio [7], we asked in the current study whether the virus can differentially alter CD4+ or CD8+ T cell proliferation and/or their apoptosis resulting in a shift in the T cell phenotypic characteristics. We found that the exposure of T lymphocytes to a naturally occurring HCV, although not necessarily molecularly evident active virus replication in these cells, can be sufficient to alter the CD4^+^ to CD8^+^ T cell ratio. This phenotypic change was due to the selective inhibition of CD4+ but not CD8+ T lymphocyte proliferation and was not related to differential apoptotic death of either T cell subset.

## Materials and methods

### Plasma-derived HCV inocula and target cells

Plasma from patients C26/F (CHC-1), C33/M (CHC-2), N28/F (CHC-3) with clinically and serologically documented CHC served as the source of naturally occurring HCV. The inocula contained HCV of different genotypes and viral loads (Table 1). CHC-1 carried a mixture of genotype 1a and 1b at 1.1 × 10^6^ virus copies, also termed virus genome equivalents (vge)/mL, CHC-2 had genotype 3a at 2 × 10^4^ vge/mL, and CHC-3 carried 2b genotype at 2.4 × 10^6^ vge/mL. These HCV plasma donors had no laboratory evidence of hepatitis B virus (HBV) or HIV type 1 (HIV-1) infection, as testing of their sera by standard clinical assays indicated, and clinically apparent chronic morbidity other than CHC.

**Table 1:** Virological characteristics of individuals with CHC providing plasma serving as HCV inocula and HCV RNA loads in T cells infected with these inocula

Inoculum	Case/Sex	HCV genotype	Plasma HCV RNA^a^ (vge/mL)	HCV RNA load in cells					
				1 d.p.i.		7 d.p.i.		10 d.p.i.	
				Positive strand^a^ (vge/μg RNA)	Negative strand^b^	Positive strand^a^ (vge/μg RNA)	Negative strand^b^	Positive strand^a^ (vge/μg RNA)	Negative strand^b^
CHC-1	C26/F	1a/1b	1.1 × 10^6^	8777	neg	1290	pos	1010	pos
CHC-2	C33/M	3a	2 × 10^4^	746	neg	204	neg	neg	NT
CHC-3	N28/F	2b	2.4 × 10^6^	<50	NT	<50	NT	<50	NT

*dpi* days post-infection, *F* female, *M* male, *NT* not tested, *pos* positive, *neg* negative

^a^Quantified by in house real-time RT-PCR

^b^Determined by the strand-specific RT-PCR/NAH

Lymphoid cells serving as targets for in vitro HCV infection experiments were isolated from a single healthy donor who had no clinical history or molecular indication of HCV exposure, as confirmed by testing for antibodies to HCV (anti-HCV) and examining serum and PBMC by highly sensitive HCV-specific RT-PCR/nucleic acid hybridization (NAH) assay (sensitivity of <10 vge/mL or <2.5 IU/mL) [2]. The donor was also serum HBV DNA and HIV-1 RNA nonreactive and had normal alanine aminotransferase (ALT) level, as determined by conventional clinical assays.

### In vitro HCV infection

*De novo* infection of lymphoid cells with HCV was carried out following the method reported before, including monocyte depletion to enhance viral replication in lymphocytes [7]. Briefly, monocyte depletion was carried out by plastic adherence for 4 h. This led to a three-fold decrease in CD14+ monocytes, as measured by flow cytometry (Additional file 1: Figure S1). Previously, we have shown that intermittent stimulation of PBMC exposed to HCV ex vivo with phytohemagglutinin (PHA) in the presence of human recombinant interleukin-2 (IL-2) leads to HCV propagation [7]. However, these conditions also augmented lymphocyte proliferation and led to a relatively high rate of lymphocyte apoptosis (data not shown). These outcomes were likely related to the repeated stimulation with PHA. To minimize this effect, which potentially masked the influence of HCV on cell proliferation and apoptosis, we stimulated lymphoid cells with PHA only once prior to infection in the current study. Thus, monocyte-depleted lymphoid cells from a healthy donor were treated with 5 μg/mL PHA (Sigma-Aldrich, Mississauga, Ontario, Canada) for 48 h [7]. Following stimulation, 1 × 10^7^ cells were exposed to 2.7 × 10^5^ vge from CHC-1 or CHC-3 or to 500 μL (1 × 10^4^ vge) of plasma from CHC-2 in 9.5 mL of culture medium. In addition, the same number of target cells was exposed to three 500-μL samples of normal healthy plasma (NHP) from 3 different healthy donors (mock infections). As another control, target cells were cultured with 9.5 mL of medium alone (NP, no plasma). In all cases, inocula or NHP were removed after 24 h and the cells washed thoroughly prior to suspension in 9.5 mL of medium, as described [7]. Cells were cryopreserved for analysis prior to and after PHA stimulation (time 0) and at 1, 4, 7 and 10 d.p.i., unless otherwise indicated. In addition, cells were collected at each of the above time points to determine cell phenotype and apoptosis (see below).

### Inhibition of HCV infection in T lymphocytes by Telaprevir

Telaprevir (TLP or VX-950), an HCV NS3-4A protease inhibitor, was purchased from Vertex Pharmaceuticals (Cambridge, Massachusetts, USA). TLP had shown capability of complete inhibition of HCV replication in *de novo* infected Molt4 T cell line [9] and naturally HCV-infected PBMC (Chen et al.*,* manuscript submitted). At concentrations equal to or below 4 μM, TLP is not toxic to human lymphocytes, as assessed before [9]. We applied the previously established treatment conditions with TLP to determine whether the shift in CD4+ T cell proliferation can be normalized in the absence of detectable virus replication in the cells previously exposed to HCV. Briefly, approximately 5 × 10^6^ cells were incubated in duplicate with CHC-1 or CHC-2 plasma under conditions described above in the presence or absence of 4 μM TLP in 0.5 % DMSO. The cells were harvested after 10 d.p.i. for evaluation of expression of HCV RNA positive and negative strands, as described above, and the CD4 and CD8 T cell frequency determined by flow cytometry. In parallel, lymphocytes exposed to the same amounts of CHC-1 or CHC-2 HCV alone and those incubated in culture medium supplemented with NHP in the absence of TLP served as infection controls.

### HCV RNA positive and negative strand detection

HCV RNA positive strand in *de novo* infected lymphoid cells as well as in patients’ plasma was determined by HCV-specific real-time RT-PCR (sensitivity 100 vge/mL) [2]. Expression of HCV RNA negative strand in T cells was detected by strand-specific RT-PCR/NAH using r*Tth* DNA polymerase as described before (sensitivity ~100 copies/μg total RNA) [2, 9].

### Determination of cell phenotype

**Fig. 1:**
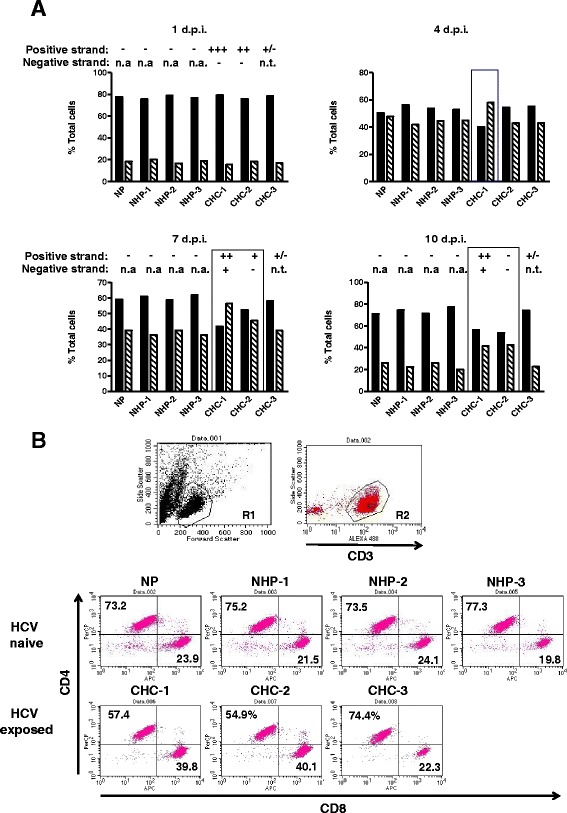
Phenotypic characterization of cultured lymphocytes after exposure to HCV. Lymphoid cells from the same healthy donor were either exposed to medium alone (no plasma, NP), normal human plasma (NHP 1–3) or HCV inocula (CHC 1–3). Cells collected at the time points indicated (d.p.i.) were stained for CD3, CD4 and CD8 or with isotype controls and analysed by flow cytometry. **a** Graphical representation of flow cytometry data showing phenotype of T cells for each infection condition tested. CD4^+^ T cells (solid black bars) and CD8^+^ T cells (hatched bars) are displayed as percentage of total CD3^+^ T cells at 4 different time points. The detection of HCV RNA positive strand was shown for 1, 7 and 10 d.p.i. For HCV RNA positive strand detection, (+++) indicates >5,000 vge/μg RNA, (++) between 500 and 5,000 vge/μg RNA, (+) between 50 and 500 vge/μg RNA, and (+/−) below 50 vge/μg RNA. For negative strand detection, (+) indicates detection and (−) no detection of the strand. Boxes indicate cultures in which the CD4^+^ to CD8^+^ ratio was altered when compared to cultures exposed to NP or NHP. n.a.- not applicable, n.t.- not tested. **b** Determination of T cell phenotype in cell cultures exposed or not to HCV and cultured for 10 d.p.i. Using forward versus side scatter, lymphocytes (gate R1) were separated from cellular debris. Lymphocytes were sub-gated on Alexa-488-positive CD3 T cells (gate R2) for enumeration of CD4^+^ and CD8^+^ T cells by detecting APC-positive CD8^+^ T cells found in the lower right (LR) quadrant and PerCP-positive CD4^+^ T cells in the upper left (UL) quadrant. Numbers in the UL and LR quadrants indicate percentages of positive cells

Phenotype of the cells exposed to HCV inocula, NHP or cultured in medium alone was assessed by incubation of 1 × 10^5^ cells per reaction with a cocktail containing an anti-CD3 monoclonal antibody (mAb) conjugated with Alexa Flour 488 (Alexa 488) (BD Pharmingen, San Diego, California, USA), an anti-CD4 mAb labelled with peridinin chlorophyll protein complex (PerCP) (BD Pharmingen) and an anti-CD8 mAb conjugated to allophycocyanin (APC) (Ebiosciences, San Diego, California, USA), an anti-CD14 mAb labeled with phycoerythrin (PE) (Ebiosciences) or with a control cocktail of the appropriate isotype mAb controls (Ebiosciences or BD Pharmingen) for 30 min at 4 °C. Using forward versus side scatter, lymphocytes were separated from debris by flow cytometric analysis (gate R1; see Fig. 1b). Then, lymphocytes were sub-gated on Alexa-488-positive CD3^+^ T cells (gate R2; see Fig. 1b) to enumerate in the next step APC-positive CD8^+^ cells located in the lower right (LR) quadrant and PerCP-positive CD4^+^ cells located in the upper left (UL) quadrant (see Fig. 1b). Quadrant markers were set up on background staining with appropriate isotype controls.

### Flow cytometry-based T cell proliferation assay

To quantify proliferation of lymphocytes exposed to HCV or NHP, cells were stained with carboxyfluorescein succinimidyl ester (CFSE), as previously reported [15]. Briefly, ~2 × 10^7^cells were incubated with CFSE (Molecular Probes, Eugene, Oregon, USA) at the pre-tested nontoxic concentration of 1 mM for 10 min at 37 °C and washed twice with 10 mL of 5 % fetal calf serum (FCS; GIBCO-Invitrogen Corporation, Auckland, New Zealand). To carry out triplicate infection with each of the HCV inocula tested, NHP or NP control, CFSE-labelled lymphocytes were suspended at 1 × 10^6^ cells in one mL of medium containing 10 % FCS in 6-well plates. As an additional control, CFSE-labelled cells in medium alone were included. Cell proliferation was measured in triplicate wells before stimulation with PHA and at 0, 1, 4, 7 and 10 d.p.i., unless otherwise stated. The data were analyzed with Cellquest Pro (Becton Dickinson, San Jose, California, USA) or ModFit LT (Verity Software House, Topsham, Main, USA) software. Using forward versus side scatter, lymphocytes (gate R1) were separated from debris. Proliferation was presented as the mean percentage of CFSE-low cells with standard error of mean (SEM) from triplicate cultures using percentage of CFSE-low cells from cultures maintained in medium alone as the baseline. In the proliferation experiments, CFSE-labelled lymphoid cells were additionally stained with a cocktail of APC-anti-CD3, PerCP-anti-CD4 and PE-anti-CD8 mAbs, as outlined above. Using forward versus side scatter, lymphocytes were separated from debris, gated on CD3^+^ T cells, and sub-gated on CD4^+^ or CD8^+^ T cells to determine the proliferation of individual T cell subsets. Mitogen non-stimulated controls were routinely included, as described above. Dilution of CFSE fluorescence in the cells was measured using the proliferation wizard module of ModFit LT software revealing daughter cell generations. Proliferation index (P.I.) was determined using non-stimulated cells to define the parent generation.

### Annexin V-PE-/7-AAD assay for detection of cell apoptosis

To determine the degree of apoptosis after exposure to HCV inocula or NHP, cells were stained with annexin V conjugated with phycoerythrin (PE), or 7-aminoactinomycin D (7-AAD), as previously established [16]. Briefly, 1 × 10^5^ cells were washed with annexin buffer containing 10 mM HEPES (Invitrogen), 5 mM NaCl, 5 mM KCl and 2 mM CaCl_2_ and centrifuged at 1,500 rpm for 10 min at 4 °C. Then, a cocktail containing 50 μg/mL of 7-AAD (Invitrogen) and 50 μg/mL of annexin-V-PE (Invitrogen) were prepared in annexin buffer. Cells were suspended in 100 μL of apoptosis cocktail and kept on ice for 30 min. After staining, cells were washed once with annexin buffer, spun down and suspended in 500 μL of annexin buffer to be analyzed by flow cytometry [16].

### Quantification of cytokine expression

In cultures in which numbers of recovered cells permitted, RNA was extracted, treated with DNase [17] and reversely transcribed [2]. Due to very limited numbers of cells available for RNA extraction, quantification of expression was feasible only for IFN-γ, IFN5-α (IFN-5α), tumor necrosis factor-α (TNF-α), and IL-2. This was done using cDNA (50 ng RNA equivalent) and amplification conditions previously reported [17]. Amplification reactions were performed using the following primers: sense primer 5′-TCAGCTCTGCATCGTTTTGG and antisense primer 5′-TGTTTTAGCTGCTGGGCACA for IFN-γ, sense primer 5′-CAGCCTGAGTAACAGGAGGA and antisense primer 5′-GCAGATGAGTCCTTTGTGCT for IFN-5α, sense primer 5′-TCTTCTCGAACCCCGAGTGA and antisense primer 5′-CCTCTGATGGCACCACCAG for TNF-α, and sense primer 5′-CCCAAGAAGGCCACAGAACT and antisense primer 5′-TGCTGATTAAGTCCCTGGGTCTTA for IL-2. Expression of the genes was normalized to β-actin.

### Statistical analysis

Data were analyzed and differences between HCV-exposed cultures and those not exposed to virus were determined using GraphPad Prism 4 software (GraphPad Software Inc., San Diego, California, USA). Statistical significance was evaluated by two-tailed Mann–Whitney test. *P* values equal to or lower than 0.05 were considered as significant.

## Results

### HCV expression in de novo infected lymphoid cells

HCV RNA positive strand was detected in T cell cultures following exposure to all three inocula tested (Table 1), but not in those exposed to NHP or medium alone (Fig. 1). The level of HCV RNA detection varied depending on inoculum and time of cell collection. HCV RNA load ranged from <50 to 8.7 × 10^3^ vge/μg total RNA (Table 1). HCV RNA negative (replicative) strand was detected at 7 and 10 d.p.i. in cultures exposed to CHC-1 inoculum that also gave the highest level of HCV RNA positive strand expression among the inocula tested.

### T cell phenotype after exposure to HCV

In preliminary experiments and in the study previously reported [7], cells exposed to HCV were maintained in culture in the presence of IL-2 under alternating stimulation with PHA. After 14 days of culture, more than 98 % cells were CD3-reactive and CD8^+^ T cells were evidently more prevalent than CD4^+^ T cells in cultures exposed to HCV [7]. Similarly, the current study, exposure of normal lymphoid cells to 2 of 3 HCV inocula tested, i.e.*,* CHC-1 and CHC-2, resulted in a change in the CD4+ to CD8+ T cell ratio in that CD8^+^ T cells became more prevalent compared to cultures exposed to NHP or cultured in medium alone (Fig. 1). Figure 1a shows relations between numbers of the CD4^+^ and CD8^+^ T cells in cultures exposed to HCV, NHP or medium alone during the 10-d.p.i. investigation period. Thus, at 1 d.p.i. the ratio of CD4^+^ T to CD8^+^ T cells was approximately 4.4 to 1 (with CD8^+^ T cells taken as 1) and was not noticeably influenced by whether the cultures were exposed or not to HCV. At 4 d.p.i., the ratio of CD4^+^ to CD8^+^ T cells changed on average to 1.2 to 1, with the exception of culture exposed to CHC-1 inoculum in which CD8^+^ T cells were enriched and the CD4+ to CD8+ ratio shifted to 0.6 to 1. At 7 d.p.i., a change in the T cell subset distribution was also observed for cells inoculated with CHC-2. At this time point, the CD4^+^ to CD8^+^ T cell ratio for CHC-1 was 0.6 to 1 and for CHC-2 1.1 to 1, while for CHC-3, NHP-1, NHP-2, NHP-3 and NP the ratio was close to 1.5 to 1. At 10 d.p.i., the CD4^+^ to CD8^+^ ratio for T cells exposed to CHC-1 and CHC-2 were 1.3 to 1 and 1.2 to 1, respectively, while the control cultures and that exposed to CHC-3 had CD4^+^ to CD8^+^ ratios of approximately 3.5 to 1 (Fig. 1a). Thus, in contrast to CHC-1 and CHC-2, CHC-3 inoculum had no effect on T cell subset phenotype and behaved, in this regard, as control NHP. This inoculum induced a very low, unquantifiable level of HCV RNA in lymphocytes throughout the culture period (Table 1 and Fig. 1a), which may suggest very limited uptake of the virus carried in this inoculum by PBMC. Taken together, these findings suggested that a change in T cell subset distribution could be related to the cellular level of HCV RNA expression.

### Apoptosis of lymphoid cells following exposure to HCV

**Fig. 2:**
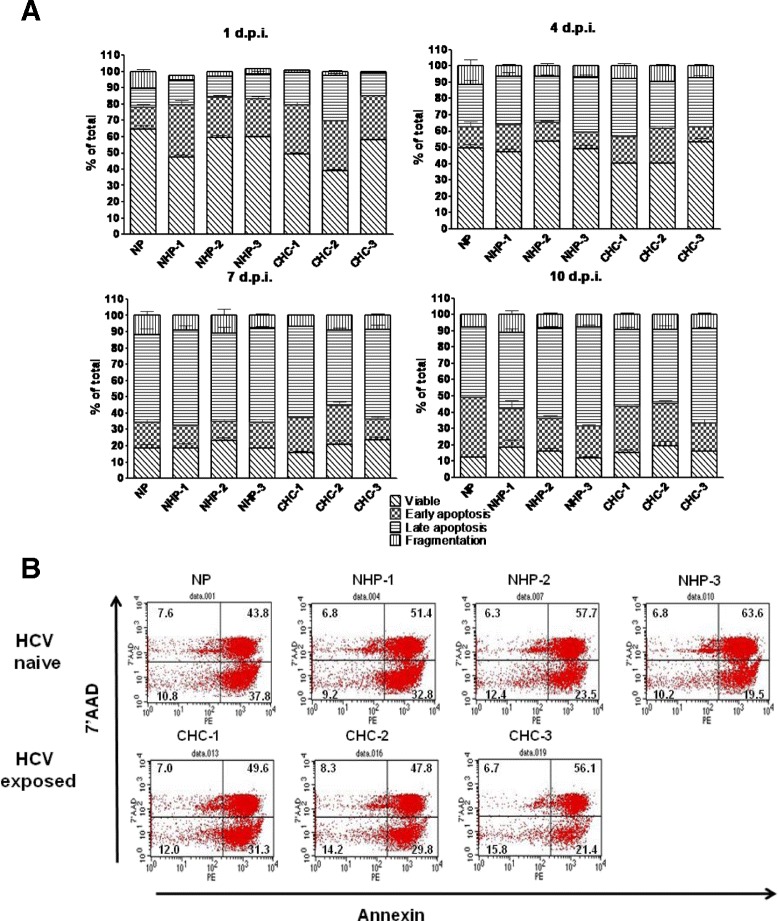
Quantification of lymphoid cell apoptosis by annexin-PE/7-AAD flow cytometry assay. **a** Graphical representation of degree of apoptosis detected in PHA-treated lymphoid cell cultures and exposed to NP, NHP 1–3 or CHC 1–3. Bars indicate the percentage of total cells in each phase of apoptosis. Graphs present data from triplicate experiments and are shown as mean ± SEM at 1, 4, 7 and 10 d.p.i. Bars with diagonal lines represent viable cells, checkered bars, early apoptotic cells, bars with horizontal lines, late apoptotic cells and vertical lines, necrotic cells. **b** Flow cytometric determination of stage of apoptosis and percentage of T cells in cultures exposed to NP, NHP or HCV at 10 d.p.i. after staining with annexin-PE and 7-AAD. Each quadrant represents a different phase of apoptosis: lower left (LL), viable cells; lower right (LR), early apoptotic cells; upper right (UR), late apoptotic cells and upper left (UL), necrotic cells. Numbers indicate percentages of cells in each quadrant

To determine whether the observed enrichment in CD8^+^ T cells after exposure to HCV was due to enhanced apoptosis of CD4^+^ T cells, the degree of lymphoid cell death was determined in triplicate cultures after exposure to a given HCV inoculum or NHP. As shown in Fig. 2, at the time points examined, the degree of apoptosis was approximately similar and not noticeably related to whether the cells were cultured in the presence of medium alone, NHP or HCV (Fig. 2a and b). Thus, under the conditions tested, the virus did not increase the rate of T cell apoptotic death. To assess whether the alteration in phenotype may result from selective apoptosis of CD4^+^ or CD8^+^ T cells, the extent of apoptotic death of these two subsets was examined separately. We found that exposure to HCV did not differentially alter apoptosis of these cells (data not shown).

### T cell proliferation after exposure to HCV

**Fig. 3:**
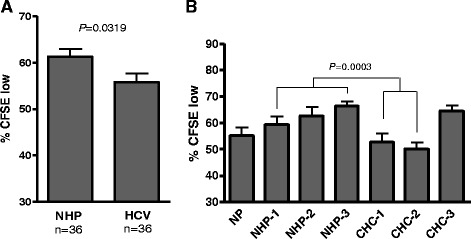
Measurement of T cell proliferation after exposure to HCV or control normal human plasma. **a** Overall comparison of proliferation in total T cell population exposed to NHP 1–3 or CHC 1–3. The data represent the mean percentage CFSE-low cells measured in triplicate cultures at 4 time points throughout the culture period (also see Additional file 2: Figure S2 for details on identification of CFSE-low cells). **b** Graphical representation of lymphoid cell proliferation after exposure to HCV inocula or NHP. Proliferation represented by the mean percentage CFSE-low and SEM measured in triplicate at 1, 4, 7 and 10 d.p.i

To determine the rate of T cell proliferation after exposure to HCV or NHP, triplicate cell cultures were evaluated using the flow cytometry-based CFSE proliferation assay. In preliminary experiments, T cells which had been stimulated more than once with PHA proliferated rapidly irrespective of HCV exposure (data not shown). However, in the current study, where cells were activated only once with mitogen prior to HCV exposure, the infection resulted in significant (*P* = 0.03) inhibition of proliferation in the total T cell population (Fig. 3a and Additional file 2: Figure S2). Upon further examination, it was established that CHC-1 and CHC-2 inocula inhibited T cell proliferation, while CHC-3 as well as NHP1-3 did not exert any remarkable anti-proliferative effect (Fig. 3b and Additional file 2: Figure S2).

### Proliferation of T cell subsets in cultures exposed to HCV

**Fig. 4:**
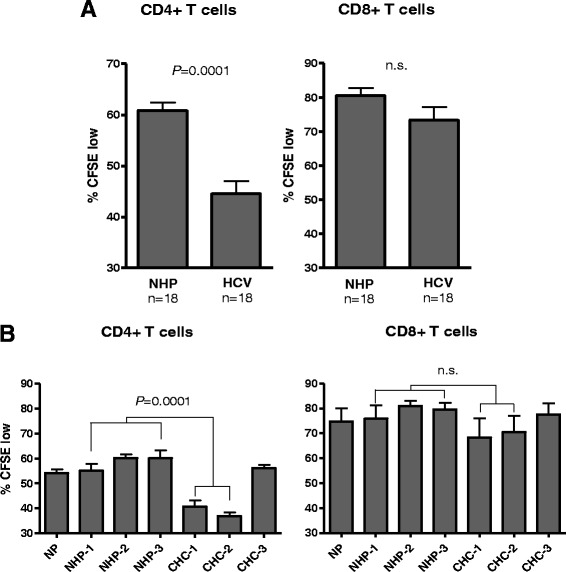
CD4^+^ and CD8^+^ T cell proliferation rates after exposure to HCV. Lymphoid cells from the same healthy donor were stained with CFSE, PHA-stimulated and infected as described in Materials and Methods to measure proliferation of CD4^+^ and CD8^+^ T cell subsets. **a** Graphical representation of proliferation of T cell subsets after exposure to either NHP or HCV inocula. Proliferation represented by the mean percentage CFSE low ± SEM was measured at 4, 7 and 10 d.p.i. **b** The rates of T cell subset proliferation in cultures exposed to individual HCV inocula or NHP. Proliferation is represented by the mean percentage CFSE-low ± SEM measured at 3 different time points during the 10-d.p.i. culture

To evaluate whether the observed inhibition in proliferation of the total T cell population could be limited to a particular T cell subset, the proliferation rates of CD4^+^ and CD8^+^ T cells were measured. When comparing cultures exposed to HCV to those treated with NHP, a significant (*P* = 0.0003) decrease in CD4^+^ T cell proliferation was found (Fig. 4a). In contrast, the proliferation of CD8^+^ T cells was not affected regardless of whether the cells were exposed to HCV or NHP. Upon further evaluation, it became apparent that CHC-1 and CHC-2 inocula caused a significant decrease in the proliferation of CD4^+^ T cell compared to the cells exposed to CHC-3 inoculum or NHP (Fig. 4b). Overall, a statistically significant decrease in the CD4+ T cell proliferation rates was seen in cultures which readily expressed HCV RNA, while no difference in CD8^+^ T cell proliferation was seen between the cultures exposed to HCV inocula or NHP (Fig. 4b).

### CD4+ and CD8+ T cell frequencies in HCV-exposed cultures treated with TLP

**Fig. 5:**
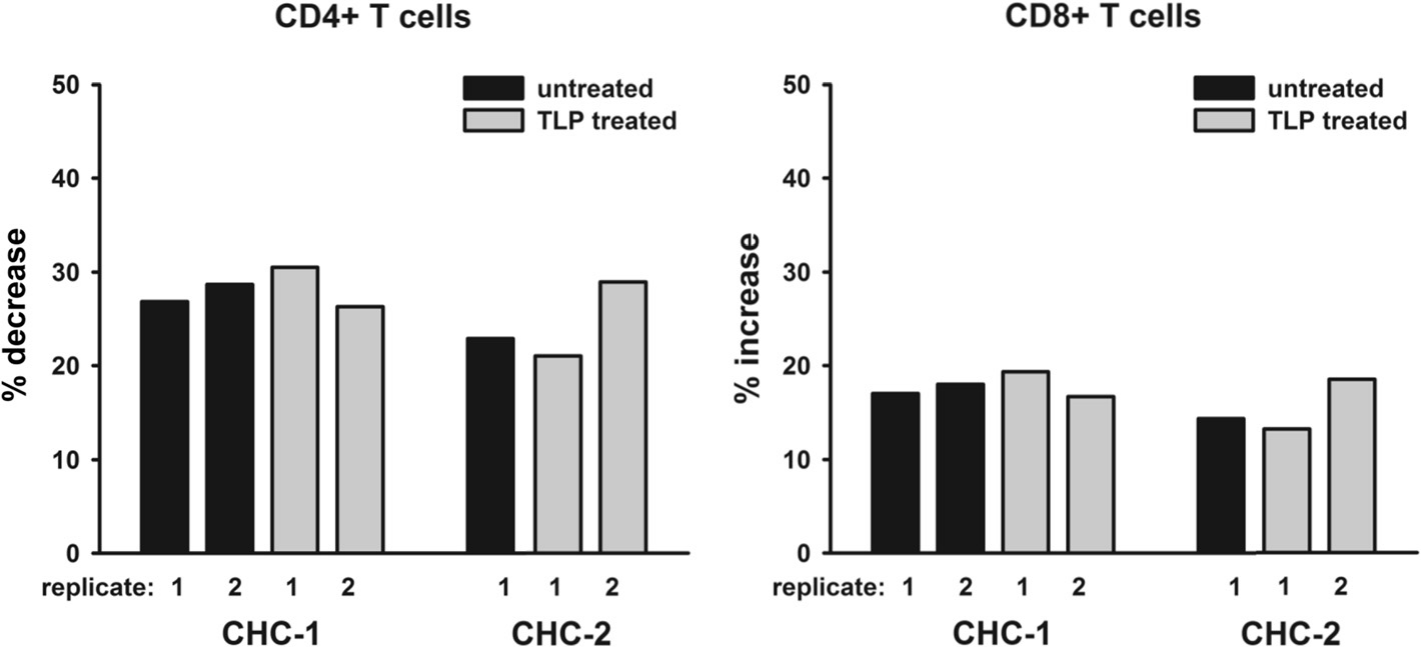
Phenotypic characterization of lymphocytes after exposure to HCV in the presence or absence of TLP. Lymphoid cells from a healthy donor were exposed in duplicate to CHC-1 or CHC-2 or to normal healthy human in the absence (untreated) or presence of TLP (TLP treated). Cells were collected at 10 d.p.i., stained for CD3, CD4 and CD8 or with isotype controls, and analyzed by flow cytometry. Percentage CD4+ T cell decrease and percentage CD8+ T cell increase was calculated based on CD4+ and CD8+ T cell frequencies (taken as 100 %) in control cultures exposed to normal human plasma

To examine the impact of HCV replication on the relative CD4+ T cell decrease and CD8+ T cell enrichment observed in earlier experiments, HCV-exposed PBMC were cultured in the presence or absence of TLP for 10 d.p.i. As expected, TLP treatment led to undetectable HCV RNA positive and negative strands in cultures exposed to CHC-1 (data not shown). For PBMC exposed to CHC-2, HCV RNA negative was undetectable either with or without TLP treatment, which was expected considering the data previously obtained (see Fig. 1). Interestingly, TPL treatment did not impact the relative decrease in the CD4+ T cell frequency caused by exposure to CHC-1 and CHC-2 inocula (Fig. 5). This suggests that uptake of virus alone, in the absence of detectable active replication, might be sufficient to impair CD4+ T cell proliferation. This may explain why we observed decreased CD4+ T cell proliferation after exposure to CHC-2 in the absence of detectable replication.

### Cytokine expression in T cell cultures exposed to HCV

To recognize the effects of HCV on the T cell cytokine expression, levels of IFN-γ, TNF-α, IFN-α5 and IL-2 mRNA were quantified in the cells treated with HCV inocula or control NHP. Due to very limited amounts of RNA, evaluation of these 4 cytokines in all cell cultures at different time points was not always feasible. In cells stimulated with PHA for 48 h prior to infection, there was an evident upregulation in IFN-γ transcription (data not shown). After PHA stimulation, IFN-γ induction achieved a peak at 1 d.p.i. in cultures exposed to 2 of 3 NHP and to all 3 HCV inocula tested. Then, the cytokine expression declined in the cultures treated with HCV but not in those exposed to NHP. Nonetheless, differences in the IFN-γ mRNA level between the cultures exposed or not to HCV did not achieve statistical significance at any of the time points examined. For TNF-α, the cytokine expression sharply declined to undetectable level at 1 d.p.i. in cultures exposed to CHC-1 and CHC-2, but not in those exposed to NHP 1–3 or CHC-3 inoculum. It is of note that cultures which lost temporarily TNF-α transcription were those in which the highest levels of cellular HCV RNA were detected (Table 1). Further, IFN-α5 expression was not detected in cells either exposed to HCV or NHP. In regard to IL-2, the cytokine mRNA level tended to be upregulated at 1 d.p.i. in cultures exposed to NHP-1 and NHP-2, but not in those treated with HCV. At later time points, IL-2 transcription level progressively declined in cultures exposed to HCV inocula but not in those treated with NHP, although the difference was statistically insignificant at the end of follow-up (10 d.p.i.).

## Discussion

In this study, we examined the effect of authentic HCV on the proliferative capacity, apoptosis and phenotype of T lymphocytes in primary cultures. Exposure of lymphoid cells to 2 of 3 HCV inocula caused a decrease in the frequency of CD4+ T cells compared to virus-untreated cultures with a relative increase in CD8+ T cells. This relative CD8^+^ T cell enrichment was a result of a significant reduction in CD4^+^ T cell proliferation compared to the cultures exposed to normal healthy plasma. On this note, the effect appeared to correlate with HCV cell uptake, since the shift in the CD4^+^ to CD8^+^ ratio was highest in the cultures in which virus exposure lead to quantifiable levels of HCV RNA positive strands and, in the case of CHC-1, negative strands in test cells (Table 1). This was consistent with the observation that in cultures exposed to CHC-1 inoculum, which induced the highest levels of HCV RNA positive strand and detectable HCV RNA negative strand, the inhibition in CD4^+^ T cell proliferation appeared earlier, i.e.*,* 4 d.p.i. In the cells exposed to CHC-2 inoculum, T cell proliferative and phenotypic changes were seen in the absence of detectable HCV replicative intermediate and after 10-fold lower multiplicity of infection compared to cells exposed to CHC-1. In the case of CHC-3, while the plasma viral load was higher than CHC-2 and comparable to CHC-1, there did not appear to be much virus uptake by cells, as indicated by undetectable HCV RNA by our high sensitivity assays. This may suggest that the amount of virus taken by cells exposed to CHC-3 was minuscule. HCV genotype may also play a role in the anti-proliferative effects observed, as CHC-1 carried an HCV genotype 1a/1b mix, CHC-2 genotype 3a, and CHC-3 genotype 2b. It is of note that there was no impact of TLP treatment on the change in the CD4+ and CD8+ T cell frequency, which suggests that exposure to HCV and uptake of the virus by cells rather than replication itself may be driving these phenotypic changes (Fig. 5).

Previously, infection of T cell lines and primary T cells with laboratory-derived HCV clones led to the impairment of T cell proliferation and IFN-γ production [4, 14]. In the current study, we show an anti-proliferative effect using patient-derived HCV as a virus source. The biological impact of HCV lymphotropism on immune cell function is a topic that is still not well understood. However, it was shown that HCV infection can led to disruption of immunological activity of different types of immune cells, including T cells, B cells, dendritic cells, monocytes and macrophages [4, 14, 18, 19].

In cultures exposed to CHC-3 inoculum, the level of HCV RNA positive strand detected during the 10-d.p.i. culture period was unquantifiable by a real time RT-PCR assay (below 50 vge/μg total RNA). However, HCV RNA signals were detected, as confirmed by NAH analysis of amplicons produced by real-time RT-PCR (data not shown). In this situation, detection of virus RNA negative strand would not be feasible, and, therefore, was not attempted (Table 1). As indicated, CHC-3 inoculum did not induce changes in T cell proliferation or phenotype, similarly as NHP-1-3, suggesting that regardless of the viral load in the inocula, if the virus cannot be taken up by cells, then the cells’ proliferation is not affected. We have previously shown that not all patient-derived HCV inocula are infectious to ex vivo pre-stimulated lymphoid cells [7, 20]. The viral and host factors determining infectivity of T cells by wild-type HCV have yet to be recognized and are under investigation in this laboratory [8, 9, 21].

In experiments leading to this study, alternating stimulation with PHA and IL-2 was applied to upregulate lymphoid cell susceptibility to HCV infection and to augment virus replication [7]. In the current work, repeated stimulation with PHA after the initial 48-h treatment was not employed to prevent excessive cell activation that may mask virus pro- or anti-proliferative or apoptotic effects. The removal of this periodical stimulation resulted in a decreased HCV replication and, therefore, a reduced detection of both virus RNA positive and negative strands. This finding is consistent with previous observations in regard to HCV replication in lymphoid cell cultures [22] and in in vitro infections with other viruses [23–25].

It is well recognized that HCV-specific CD4^+^ T cell response in individuals chronically infected with HCV is poor or absent [26]. In HCV-infected patients who transiently control viremia and have fluctuating plasma viral load, including periods of apparent HCV RNA negativity, this transient viral control is accompanied by increased virus-specific CD4^+^ T cell response [26]. In patients with CHC, T cells have been found to be impaired in the production of IFN-γ and IL-2 [12]. As well, studies investigating CD4^+^ T cell function during a symptomatic persistent HCV infection have shown a significant loss of IL-2 secreting cells compared to individuals who spontaneously resolved viremia, as well as a weak IFN-γ production by HCV-specific CD4^+^ T cells upon stimulation [27]. In the latter study, HCV-specific IFN-γ production by CD4^+^ T cells was rescued after in vitro culture with exogenous IL-2, but the effect of IL-2 on the CD4+ T cell proliferation was not measured. These findings may suggest that impaired proliferation of CD4^+^ T cells observed in our study could be due to weakened IL-2 production. However, only CD4+ T cells but not CD8+ T cells were affected. Due to limitations in cell numbers we were not able to evaluate whether there was indeed impaired IL-2 and/or IFN-γ cytokine production by in vitro infected T cells. This issue requires further investigation. Preliminary data obtained in this regard in the present study indicate that there were no significant differences between T cells exposed or not to HCV in terms of IFN-γ, IFN-5α and IL-2 mRNA expression. However, TNF-α transcription appeared to be transiently but severely down-regulated shortly (i.e., 1 d.p.i.) after exposure to CHC-1 or CHC-2. This finding warrants further examination. Nonetheless, it needs to be taken under consideration that the level of cytokine gene expression may not accurately reflect the cytokine protein production.

The results of this study should be interpreted with certain limitations taking under consideration that PBMC exposed to authentic HCV were isolated from a single healthy donor. The observations made might be limited to this particular donor and experiments with cells from other healthy individuals are needed to confirm the findings. However, we have previously reported that a comparable shift in the CD4+ to CD8+ T cell ratio also occurred after exposure to native HCV of T cells from another healthy donor [7].

Overall, our findings imply that exposure to HCV can modify the T cell phenotype due to a relative decrease in the proliferation capacity of CD4^+^ T cells. Furthermore, exposure to naturally occurring HCV in vitro did not appear to differentially augment apoptotic death of lymphocyte subsets, although apoptotic effects of a HCV clone on T cells have been described previously [18]. In this context, it needs to be determined whether primary CD4+ and CD8+ T cells derived from patients with CHC display similar characteristics. The results from the current study raise a possibility that HCV may exert a direct effect on the overall T cell phenotypic properties and, in consequence, on the function of T cells as a whole in HCV-infected patients.

## Additional files

Additional file 1: Figure S1. Monocyte depletion prior to cell infection with HCV. Monocytes were depleted from PBMC via plastic adherence for 4 h and their frequencies in total PBMC and monocyte-depleted PBMC were determined after staining with anti-CD14 antibody by flow cytometry. Additional file 2: Figure S2. Flow cytometric determination of T cell proliferation after exposure to medium alone (NP), NHP 1–3 or HCV inocula CHC 1–3 at 7 d.p.i. Using forward versus side scatter, lymphocytes (gate R1) were separated from debris. Percentage CFSE low was determined based on unstimulated control cells cultured with medium alone. Low left (LL) and low right (LR) quadrants represent cells with CFSE low (dim) and CFSE high (bright) reactivity, respectively. Dilution of CFSE fluorescence was analyzed with ModFit LT showing daughter generations. P.I. values were determined by using non-stimulated cells to define parent generation.
