# Supplementary figures and images for: Patient-derived hepatitis C virus inhibits CD4+ but not CD8+ T lymphocyte proliferation in primary T cells
Source: Virol J. 2015 Jun 19;12:93. doi: 10.1186/s12985-015-0322-4 (PMC4474354; doi:10.1186/s12985-015-0322-4)

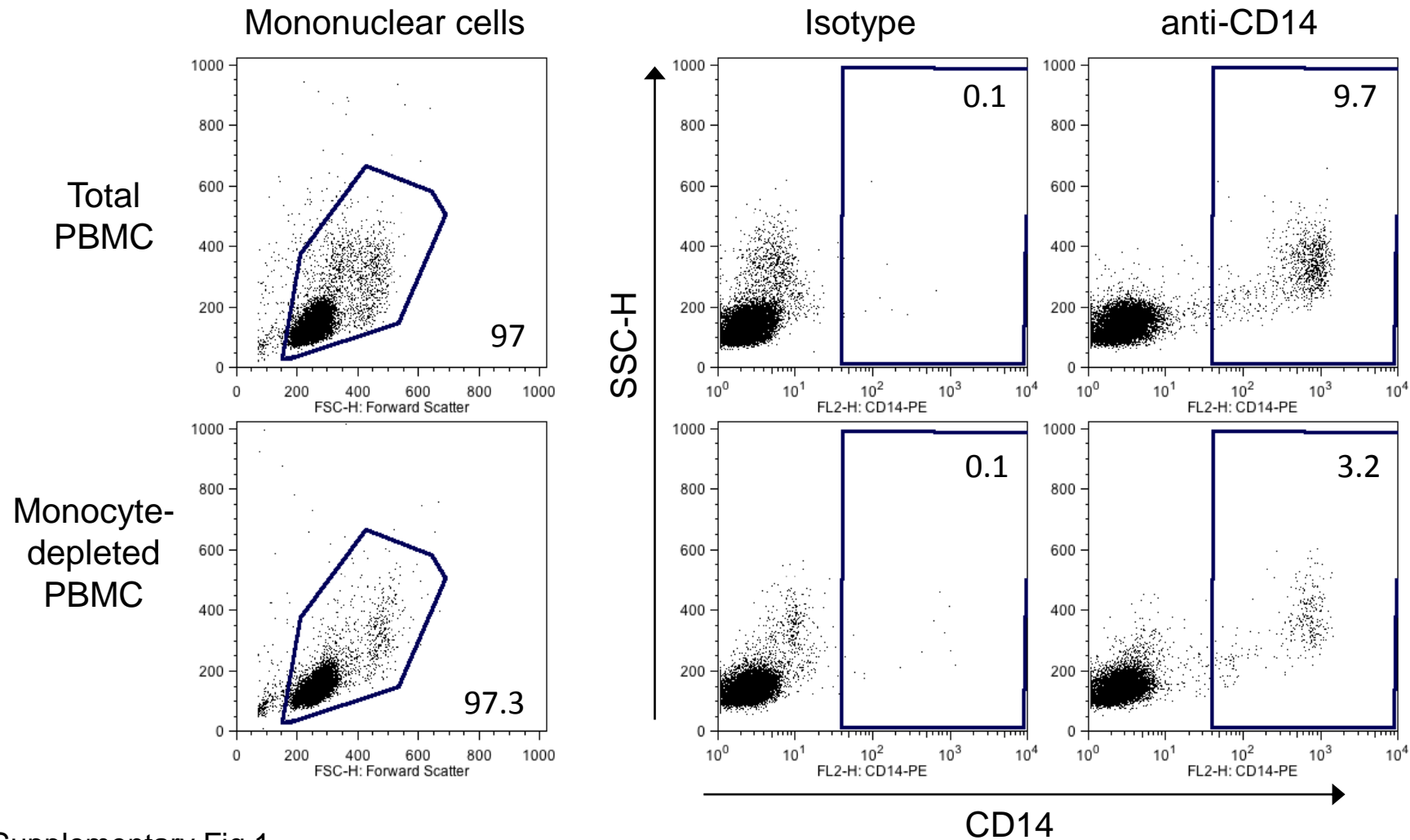

Supplementary Fig 1

Supplement: Additional file 1: Figure S1. — Monocyte depletion prior to cell infection with HCV. Monocytes were depleted from PBMC via plastic adherence for 4 h and their frequencies in total PBMC and monocyte-depleted PBMC were determined after staining with anti-CD14 antibody by flow cytometry. [file 12985_2015_322_MOESM1_ESM.pdf]

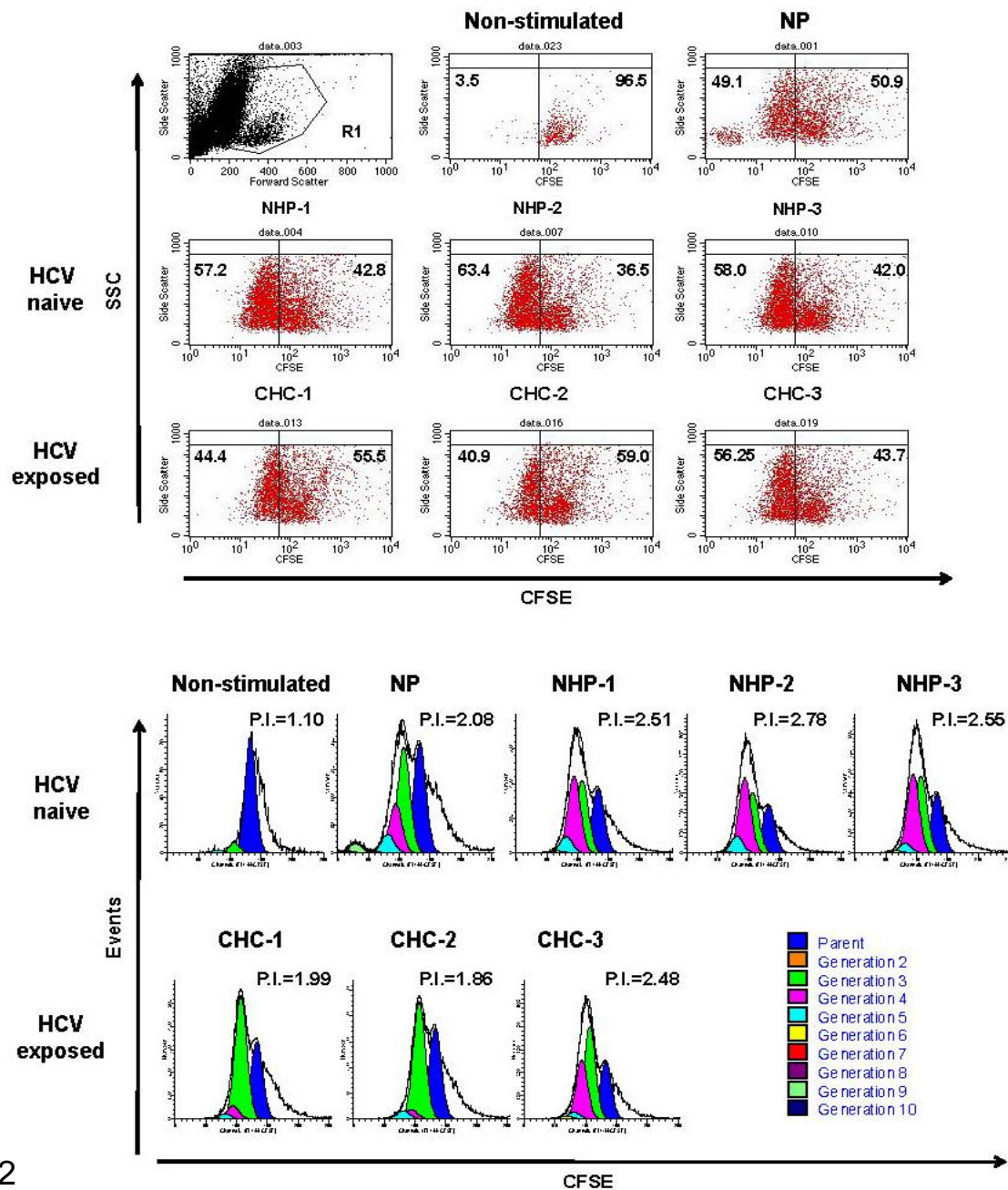

Supplementary Fig. 2

Supplement: Additional file 2: Figure S2. — Flow cytometric determination of T cell proliferation after exposure to medium alone (NP), NHP 1–3 or HCV inocula CHC 1–3 at 7 d.p.i. Using forward versus side scatter, lymphocytes (gate R1) were separated from debris. Percentage CFSE low was determined based on unstimulated control cells cultured with medium alone. Low left (LL) and low right (LR) quadrants represent cells with CFSE low (dim) and CFSE high (bright) reactivity, respectively. Dilution of CFSE fluorescence was analyzed with ModFit LT showing daughter generations. P.I. values were determined by using non-stimulated cells to define parent generation. [file 12985_2015_322_MOESM2_ESM.pdf]
